# Supplementary material for: Identification of Freezing-Responsive microRNAs and Their Targets in Chinese Jujube by Small RNA and Degradome Sequencing
Source: Int J Mol Sci. 2026 Sep 4;27(17):7912. doi: 10.3390/ijms27177912 (PMC13566890; doi:10.3390/ijms27177912)
Supplement: Supplementary file 1 [file ijms-27-07912-s001.zip › Supplementary Material S2/novel_miR_184.pdf]

Provisional ID	:	novel_miR_184
Score total	:	43.2
Score for star read(s)	:	3.9
Score for read counts	:	41.4
Score for mfe	:	0
Score for randfold	:	-2.2
Score for cons. seed	:	
Total read count	:	93
Mature read count	:	88
Loop read count	:	0
Star read count	:	5

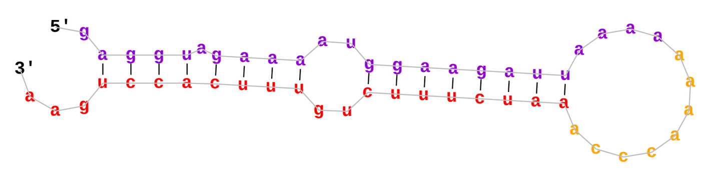

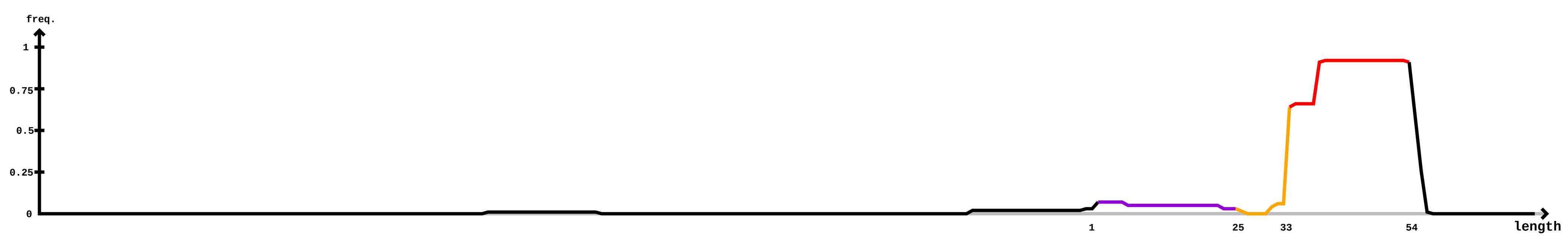

Star Mature

```
5'- aaaucgugcuaauauuaucucaugagagggacaauuuaacccgaaccauuaaaauugugacaagaaaaacgguaauccaaaggcuuuuuaauuuaauuuuuauuauuauuauuuuuuuuuuauagggagccaaaggcuuuuuuaauuugauauauauacuguuauagugaagguuggagguagaaaauggaagauuaaaaaaaacccaaaucuuucuguuucaccugaaaucucucgcuuuuucauuuuua -3' obs
    aaaucgugcuaauauuaucucaugagagggacaauuuaacccgaaccauuaaaauugugacaagaaaaacgguaauccaaaggcuuuuuaauuuaauuuuuauuauuauuauuuuuuuuuuauagggagccaaaggcuuuuuuaauuugauauauauacuguuauagugaagguuggagguagaaaauggaagauuaaaaaaaacccaaaucuuucuguuucaccugaaaucucucgcuuuuucauuuuua exp
    .(((.(((...((((((((((....)))).((((((((........)))))))))...(((((((.((((((...((((.((........)).))))...)))))).))))))))....((((((((...))))))))).....))))))...))).))).((((((((((..((((.((((..((((((((...........))))))))..))))))))..))))).)))))...........  reads  mm  sample
```

read	reads	mm	sample
...auccaaUggcuuuuuuaauu...	1	1	LT-1
...auacuguuauagugaagguuggaggu...	2	0	JCK-1
...uggagguagaaaauggaagauAa...	1	1	JCK-1
...gagguagaaaauggaagauua...	1	0	LT-1
...gagguagaaaauggaagauuaaaa...	3	0	LCK-3
...ccaaaucuuucuguuucaccugaa...	4	0	LT-3
...caaaucuuucuguuucaccugaa...	2	0	JCK-3
...aaucuuucuguuucaccugaa...	26	0	LCK-1
...aaucuuucuguuucaccugaa...	32	0	LCK-2
...aucuuucuguuucaccuga...	1	0	LCK-3
...aucuuucuguuucaccugaa...	1	0	LT-2
...uAcuguuucaccugaaau...	13	1	JT-2
...uAcuguuucaccugaaau...	12	1	JT-3
...ucuguuuUaccugaaauc...	1	1	JCK-2
